# Pentad: A reproducible cytoarchitectonic protocol and its application to parcellation of the human hippocampus

**DOI:** 10.3389/fnana.2023.1114757

**Published:** 2023-02-09

**Authors:** Emily M. Williams, Emma W. Rosenblum, Nicole Pihlstrom, Josué Llamas-Rodríguez, Samantha Champion, Matthew P. Frosch, Jean C. Augustinack

**Affiliations:** ^1^Department of Radiology, Athinoula A. Martinos Center for Biomedical Imaging, Massachusetts General Hospital, Boston, MA, United States; ^2^Department of Neuropathology, Massachusetts General Hospital, Boston, MA, United States

**Keywords:** CA1, CA2, CA3, hippocampal subfields, histology, pyramidal neurons, segmentation, subiculum

## Abstract

**Introduction:**

The hippocampus is integral for learning and memory and is targeted by multiple diseases. Neuroimaging approaches frequently use hippocampal subfield volumes as a standard measure of neurodegeneration, thus making them an essential biomarker to study. Collectively, histologic parcellation studies contain various disagreements, discrepancies, and omissions. The present study aimed to advance the hippocampal subfield segmentation field by establishing the first histology based parcellation protocol, applied to *n* = 22 human hippocampal samples.

**Methods:**

The protocol focuses on five cellular traits observed in the pyramidal layer of the human hippocampus. We coin this approach the pentad protocol. The traits were: chromophilia, neuron size, packing density, clustering, and collinearity. Subfields included were CA1, CA2, CA3, CA4, prosubiculum, subiculum, presubiculum, parasubiculum, as well as the medial (uncal) subfields Subu, CA1u, CA2u, CA3u, and CA4u. We also establish nine distinct anterior-posterior levels of the hippocampus in the coronal plane to document rostrocaudal differences.

**Results:**

Applying the pentad protocol, we parcellated 13 subfields at nine levels in 22 samples. We found that CA1 had the smallest neurons, CA2 showed high neuronal clustering, and CA3 displayed the most collinear neurons of the CA fields. The border between presubiculum and subiculum was staircase shaped, and parasubiculum had larger neurons than presubiculum. We also demonstrate cytoarchitectural evidence that CA4 and prosubiculum exist as individual subfields.

**Discussion:**

This protocol is comprehensive, regimented and supplies a high number of samples, hippocampal subfields, and anterior-posterior coronal levels. The pentad protocol utilizes the gold standard approach for the human hippocampus subfield parcellation.

## 1. Introduction

The hippocampal proper (CA1–4), the dentate gyrus (DG), and the subicular cortices [prosubiculum (ProS), subiculum (Sub), presubiculum (PreS), and parasubiculum (ParaS)] comprise the hippocampal formation. The hippocampus (CA1–4, subicular cortices) is well known for its crucial role in learning and memory (Scoville and Milner, 1957; Eichenbaum, 2004; Squire, 2004; Warren et al., 2012; Buzsáki and Moser, 2013; Rubin et al., 2014). Multiple diseases affect the hippocampus, including Alzheimer’s disease (Braak and Braak, 1991; Braak et al., 2006), epilepsy (Dam, 1980; de Lanerolle et al., 1989; Engel, 1996), schizophrenia (Heckers et al., 1998; Harrison, 2004; Zhou et al., 2008), and post-traumatic stress disorder (Bonne et al., 2008; Logue et al., 2018; Harnett et al., 2020). Further, the individual subfields of the human hippocampus have specialized and unique functions; for instance, Sub is involved in scene discrimination (Zeidman and Maguire, 2016; Hodgetts et al., 2017) while CA3/DG is relevant to pattern separation (Bakker et al., 2008; Yassa et al., 2011; Deuker et al., 2014). In addition, the subfields have unique vulnerabilities to disease. CA1, for example, is especially vulnerable to hypoxia (Ouyang et al., 2007; Bartsch et al., 2015; Butt et al., 2021) and schizophrenia (Schobel et al., 2009; Ho et al., 2017; Baglivo et al., 2018), and CA3 is especially susceptible to limbic seizures (Cherubini and Miles, 2015; Medvedeva et al., 2017). Thus, to further elucidate presumptive hippocampal subfield functions and to treat disease, it is imperative to accurately delineate subfield boundaries.

The neuroimaging field has experienced a great push forward in hippocampal subfield segmentations (Raz et al., 2015; Yushkevich et al., 2015; Adler et al., 2018; de Flores et al., 2020) and some groups have built atlases that can be used by other groups to parcellate the hippocampal subfields (Morey et al., 2009; Iglesias et al., 2015; Bender et al., 2017; Wisse et al., 2017). However, the MRI field has vastly outpaced the neuroanatomy field, and while MRI resolution has improved in the past two decades, MRI (or any neuroimaging) does not show the neurons and cytoarchitecture of the hippocampus. This lack of resolution makes it difficult to know precisely where the hippocampal subfield boundaries reside. As a result, many neuroimagers resort to grouping subfields together (Schoene-Bake et al., 2014; Iglesias et al., 2015; Parekh et al., 2015; Raz et al., 2015; Winterburn et al., 2015; Daugherty et al., 2016; Bender et al., 2017; Wisse et al., 2017; de Flores et al., 2020), or base boundaries on extrapolations rather than cytoarchitecture (Mueller et al., 2007; Wisse et al., 2012; Steve et al., 2017). Moreover, the existing segmentation protocols differ drastically among neuroimaging groups, which makes it difficult to understand individual subfield differences and to make comparisons (Morey et al., 2009; Yushkevich et al., 2015; Wisse et al., 2017).

To overcome some of the resolution issues found in MRI scans, some neuroimagers have started to use histology as the basis of their parcellation (Adler et al., 2014, 2018; Goubran et al., 2015; Peixoto-Santos et al., 2018; de Flores et al., 2020). Still, neuroanatomists differ in their segmentations. This could be due to the lack of an established and harmonized histologically based parcellation protocol (Farrell et al., 2022). While there have been detailed histology-based parcellations on human hippocampal subfields (Lorente de Nó, 1934; Rosene and Van Hoesen, 1987; Insausti and Amaral, 2004; Ding, 2013; Ding and Van Hoesen, 2015; Zilles et al., 2015; Insausti et al., 2017; Adler et al., 2018; Palomero-Gallagher et al., 2020), none have provided a structured and organized protocol. Further, histology parcellations have been highly variable and are plagued with disagreements. For instance, many studies on the hippocampal subfields do not include subfields such as presubiculum and parasubiculum (Lorente de Nó, 1934; Duvernoy, 2005; Bonnici et al., 2012; Daugherty et al., 2016; de Flores et al., 2020). Some authors include prosubiculum as a subfield (Ding and Van Hoesen, 2015; Palomero-Gallagher et al., 2020), while others do not (Insausti and Amaral, 2004; Adler et al., 2018). Additionally, multiple authors do not provide specific labeling or explicit boundary lines on histological images (Rosene and Van Hoesen, 1987; Insausti and Amaral, 2004; Duvernoy, 2005; Farrell et al., 2022). Some histology publications also do not illustrate more than one example of a parcellated case (Insausti and Amaral, 2004; Adler et al., 2014), thus not giving examples of the inevitable variability that comes with working with human brain tissue. The question of how to parcellate becomes too abstract without multiple examples. Lastly, previous approaches have focused on whole layers within the hippocampus (not cells), imprecise definitions, or not collectively evaluated multiple traits. Thus, the hippocampal field has yet to reliably delineate subfield boundaries using histology in the human brain.

There is a need for a harmonized, manual histology parcellation protocol that incorporates existing work, settles the disagreements, and provides clarity and guidance in subfield parcellation using multiple cases. Wisse et al. (2017) published a report on the necessities of such a protocol. Notedly, Ding and Van Hoesen (2015) and Palomero-Gallagher et al. (2020) have modernized the subfield parcellation protocol with boundary lines and multiple neuroanatomical markers. Adler et al. (2018) and de Flores et al. (2020) have continued this advance in producing the human hippocampal subfield parcellations in MRI models. Presently, we sought to harmonize previously found subfield markers and define novel markers (i.e., collinearity) to establish a detailed, histology-based parcellation protocol and to apply this protocol to 22 cases. This protocol’s ability to withstand individual, subfield, and anterior-posterior variability shows that this parcellation method offers clarity and consistency for neurohistology of the human hippocampus. It may ultimately provide a positive impact on the neuroimaging community by building cohesion. These findings provide a parcellation guide for the hippocampal subfields using the gold standard histology (not MRI) approach to define subfield boundaries.

## 2. Materials and methods

### 2.1. Tissue samples

Twenty-two brain hemispheres were collected by the Massachusetts General Hospital Autopsy Service and were fixed with 10% formalin for at least 2 months. Consent to autopsy was obtained prior to death. All experiments abided by guidelines approved by Internal Review Board at the Massachusetts General Hospital. Table 1 lists the demographics for all cases studied. All brain tissue were screened and diagnosed by two neuropathologists to control for comorbidities (MF and SC). The sex ratio was 8 females and 12 males (two cases did not have sex demographics), and ages ranged from 45 to 84 years old. There were 13 left hemispheres and 9 right hemispheres in the dataset. Postmortem intervals (PMI) before fixation were less than 24 h, except for one case that was 48 h. The tau antibody, CP13 (gift from Dr. Peter Davies), was used to assess neurofibrillary (tau) tangle severity and Braak and Braak staging (MF and JA) (Braak and Braak, 1991, 1995; Braak et al., 2006). The Braak and Braak staging of the sample set were *n* = 8 normal controls (NC), *n* = 5 Braak and Braak stage I cases, and *n* = 9 Braak and Braak stage II cases. In the Braak and Braak staged I and II cases, the tau accumulation was primarily limited to the perirhinal and entorhinal cortex (Braak and Braak, 1991), and contained minimal, if any, neuropathologic changes in the hippocampus.

**TABLE 1:** Demographic information for cases used in this study.

Case ID	Age (years)	Sex	Hemisphere	Cause of death	PMI (hrs)	Brain weight (g)	Braak stage
1	67	M	RH	N/A	12	1,199	C
2	N/A	N/A	RH	N/A	<24	N/A	C
3	68	M	RH	Malignant mesothelioma	<24	1,320	C
4	45	F	LH	Ischemic liver	24	1,215	C
5	45	F	LH	Lung disease	<24	1,411	C
6	49	M	LH	End stage liver cirrhosis	3	1,300	C
7	67	M	RH	Lung cancer	<48	1,380	C
8	61	M	RH	Stroke	23	1,310	C
9	60	M	RH	Aortic dissection	14	1,414	BBI
10	68	M	RH	Acute cardiac death	<24	1,595	BBI
11	59	M	LH	Liver failure	20	1,320	BBI
12	79	M	LH	Surgery complications	15	1,200	BBI
13	73	F	RH	Aortic dissection	23	1,356	BBI
14	59	F	LH	Heart failure	<24	1,402	BBII
15	73	F	LH	Hemorrhagic telangiectasia	<24	1,142	BBII
16	N/A	N/A	LH	N/A	<24	N/A	BBII
17	60	M	RH	Liver failure	<24	1,166	BBII
18	84	F	LH	Heart failure	<24	1,221	BBII
19	74	F	LH	Coronary disease	24	1,060	BBII
20	78	M	LH	Liposarcoma	24	1,320	BBII
21	60	F	LH	Pancreatic cancer	2	1,328	BBII
22	75	M	LH	Vascular disease	24	1,187	BBII

Sorted by Braak and Braak stage severity. Braak stage, Braak and Braak staging; C, control; g, grams; hrs, hours; LH, left hemisphere; N/A, not available; PMI, post-mortem interval; RH, right hemisphere.

### 2.2. Blocking and sectioning procedures

Hemispheres were stored in periodate-lysine-paraformaldehyde at 4°C, then medial temporal lobes were dissected from the surrounding temporal lobe perpendicular to the long axis of the hippocampus, following previously published procedure (Adler et al., 2014; Steve et al., 2017; de Flores et al., 2020). The temporal lobe blocks were ∼5 cm in length, which included the hippocampal head and body. Blocked tissue was incubated in cryoprotectant (20% glycerol, 2% dimethyl sulfoxide) for at least 1 month to ensure tissue was well protected and prepared for sectioning. Samples were manually and serially sectioned in the coronal plane using a sliding freezing microtome (Leica SM2000R, Leica Biosystems Inc., Buffalo Grove, IL, USA) at 50 μm, and every section was collected and stored in cryoprotectant at −20°C.

### 2.3. Histology

Every 10th tissue section was selected, rinsed in phosphate buffer solution to remove cryoprotectant, and manually mounted on gel coated glass slides. Approximately 40 sections from each case underwent the Nissl staining. Mounted sections were dried for 24 h, then stained for thionin (Nissl) (Zilles et al., 2002; Augustinack et al., 2005). The staining procedure was as follows: (1) defat [100% ethanol: chloroform (1:1)], (2) rinse [50% ethanol, then twice distilled water (ddH_2_O)], (3) pre-treatment [acetic acid: acetone: ddH_2_O: 100% ethanol (1:1:1:1)], (4) staining (5% aqueous thionin, sodium acetate stock, and acetic acid stock), and (5) differentiation (70% ethanol and glacial acetic acid). The slides were then dehydrated in ascending concentrations of ethanol, dipped in xylene to eliminate remaining water. Finally, slides were coverslipped using Permount (Fisher Scientific, Hampton, NH, USA).

### 2.4. Slide digitization and analysis

All Nissl stained tissue were examined using an Olympus BH-2 double headed microscope (Precise Instrument, Hansen, MA, USA), a Nikon SMZ1000 (Nikon, Japan) parcellation scope with a Fiber-Lite illuminator (MVI, Avon, MA, USA), and a Keyence BZX800 (Keyence, Japan). The double-headed microscope was used to examine the cytoarchitecture closely and allowed for discussion among raters (EW and JA) when evaluating. Subfield boundaries were drawn with ultra-fine Sharpie pen under the dissecting scope (Nikon SMZ-1000) and Dolan-Jenner light source (MVI, Avon, MA, USA). Slides were digitized at 4× using the Keyence microscope. The subfield boundaries were transferred to the 4× images of the slides using GIMP (open-source visualization software in Unix operating systems) to keep a digital record of the boundary work.

### 2.5. Nine hippocampal anterior-posterior levels

**FIGURE 1:**
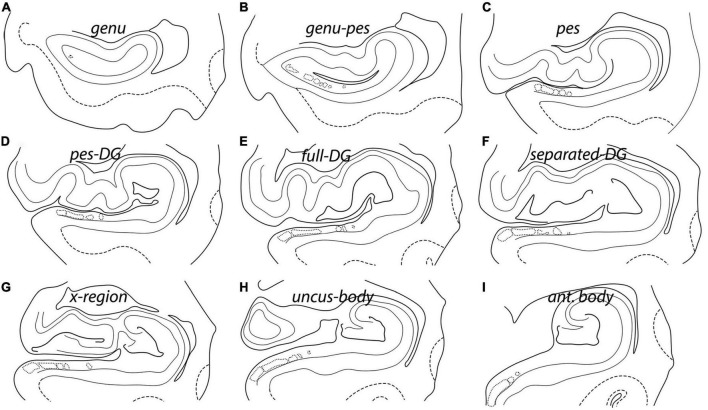
Nine rostrocaudal hippocampal levels represented by line drawing schemata in the coronal plane. Each level shows different landmarks that makes it unique. Accordingly, each anterior-posterior level has been given a unique name: **(A)** Genu, **(B)** genu-pes, **(C)** pes, **(D)** pes-DG, **(E)** full DG, **(F)** separated DG, **(G)** x-region, **(H)** uncus-body, and **(I)** anterior body. The solid black lines represent the pial tissue surface and the dentate gyrus. The gray lines denote the gray-white matter boundaries, while the dashed lines indicate gray-white matter boundaries in the entorhinal cortex. The lightest gray lines depict the pyramidal layer, and dotted lines denote the presubicular clouds, LPE, and ParaS.

The anatomy of the hippocampus and intertwined DG is complicated and undergoes many structural changes as it progresses from anterior to posterior in the coronal plane. To best represent all morphological changes of the hippocampus and its subfields across the entirety of the anterior-posterior axis, we selected nine levels to investigate. The nine levels consist of (1) genu (Rosene and Van Hoesen, 1987), (2) genu-pes, (3) pes, (4) pes-DG, (5) full DG, (6) separated DG, (7) x-region (de Flores et al., 2015), (8) uncus-body, and (9) body (Figure 1). The term genu represents the most anterior level and is relatively small (no pes) (Figure 1A). The genu-pes level occurs when at least one pes (digitations) emerges, but not all have fully formed yet (Figure 1B). At the pes level, the dorsal hippocampal ridge forms at least two pes (resembling toes) (Figure 1C). Notably, the DG is still posterior to this level and is not present in the genu, genu-pes, or pes levels. The next level is the pes-DG, wherein the DG begins anteriorly usually with a thin strip of DG (Figure 1D). When the DG occupies its widest stretch from medial to lateral, that coronal level is named the full-DG (Figure 1E). The next level is separated-DG and occurs when the anatomical structures – the HP body and gyrus intralimbicus (Gloor, 1997; Insausti et al., 2019) – separate but the pyramidal layer is still fully connected (Figure 1F). Following this level is the x-region (de Flores et al., 2015), which refers to the level where the DG has been split in two parts, and the pyramidal layer begins to separate into two disparate portions (Figure 1G). The uncus-body (gyrus intralimbicus) level results once the medial portion detaches from the hippocampal body completely (Figure 1H). Finally, the most posterior level is hippocampal body, where the uncinate gyrus and hippocampal head has ended (Figure 1I).

### 2.6. Pentad protocol – The five traits: Chromophilia, neuron size, packing density, clustering, and collinearity

We collated and expanded upon cytoarchitectural traits from the existing subfield and boundary work (Lorente de Nó, 1934; Rosene and Van Hoesen, 1987; Zilles et al., 2002; Insausti and Amaral, 2004; Ding, 2013; Adler et al., 2014; Ding and Van Hoesen, 2015; Insausti et al., 2017; Steve et al., 2017; de Flores et al., 2020; Palomero-Gallagher et al., 2020). Table 2 summarizes several seminal previous publications and details the histologic characteristics noted for each respective study. We expanded on these traits and established an entirely new trait, collinearity, to distinguish CA2 from CA3, thus creating a regimented protocol with five weighted traits applicable to all subfields. This protocol, termed the pentad protocol, describes cellular, and architectural traits, but more importantly, provides a flexible approach that weighs each trait for parcellation. The pentad protocol defines five characteristics: chromophilia, neuron size, packing density, clustering, and collinearity (Table 3). Table 3 summarizes the cytoarchitectural definitions of these five traits. First, chromophilia (“stain loving”) refers to the overall intensity of the staining in neurons: a chromophilic neuron shows a rich, dark hue in Nissl stained tissue due to numerous ribosomes in neurons (for review, Gomes, 2019). Second, neuronal size describes general size of the neuronal soma. Third, packing density refers to the overall closeness of neurons. Clustering is the fourth trait, which evaluates whether the packing density of the layer is homogenous or heterogeneous (i.e., distinct clustering of neurons within the cell layer). Fifth and last, collinearity describes the orientation of neurons within the subfield.

**TABLE 2:** Summary of previous seminal publications and their main observations (rows) on the histological characteristics of each hippocampal subfield (columns).

	ParaS	PreS	Sub	ProS	CA1	CA2	CA3	CA4
Lorente de Nó (1934) Golgi	**	**	5 layers, has both triangular shaped pyramids and “globular or polygonal” pyramids	No stratum radiatum or lacunosum, identifiable using silver stains	Smallest neurons of the CA fields, very thin axons, presence of stratum radiatum	Very large neurons, no thick thorns on dendrites, smallest pyramidal layer	Very large neurons; mossy fibers	Modified pyramids, non-uniform structure, no stratum radiatum
Rosene and Van Hoesen (1987) Nissl, Timm’s, Ach	**	**	Wide pyramidal layer, much bigger neurons than CA1 and ProS	Small neurons, clustering of superficial cells, no stratum oriens	Wide pyramidal layer, sparsely packed, lightly stained, slightly smaller than CA3 neurons	Large and darkly stained neurons; no mossy fibers	Tightly packed, darkly stained neurons; mossy fibers	Modified pyramids, mossy fibers
Insausti and Amaral (2004) Nissl, Timm’s	Single cell layer, larger and sparser pyramidal neurons than PreS	Single cell layer, small and modified pyramidals, presence of clouds	Larger and more sparsely packed neurons than CA1, thicker pyramidal layer	Not included	Heterogenous staining properties, wide pyramidal layer, has stratum radiatum	Large neurons, more densely packed than CA3	Largest and darkly stained neurons of HP, mossy fibers	Not included
Duvernoy (2005) India ink, Bodian’s silver	**	Small, superficial clouds	No stratum radiatum	**	Small neurons and sparsely packed	Large and densely packed neurons, narrow pyramidal layer	Mossy fibers, less densely packed than CA2	Large, sparsely packed neurons. Not many neurons
Ding and Van Hoesen (2015) Nissl, NeuN, parvalbumin, calbindin	Larger neurons than PreS	Narrow molecular layer, LPE	Large, neurons, heterogenous staining	Small and densely packed neurons	Lightly stained, small	Large neurons, less stained than CA3	Mossy fibers, large neurons	Not included
Adler et al. (2014) Luxol fast blue + cresyl violet	Larger neurons than PreS	Small neurons	Pyramidal layer has striated appearance	Not included	Sparsely packed	Large, densely packed	Thin pyramidal layer	Not included

Stains that were used in each report are listed below the authors and publication year. Asterisks (**) represent subfields that were listed in a figure in a manuscript, but no information was provided in the text on the subfield or its characteristics. Rosene and Van Hoesen (1987), Duvernoy (2005), and Insausti and Amaral (2004) represent books or book chapters, while Ding and Van Hoesen (2015), Adler et al. (2014), and Lorente de Nó (1934) serve as primary papers.

**TABLE 3:** Pentad protocol’s cytoarchitectural traits, their definitions, and where they are most and least applicable.

	Chromophilia	Neuron size	Packing density	Clustering	Collinearity
Definition	Staining intensity; darkly stained neurons, if lacks chromophilia then lightly stained.	General size of the neuronal soma; ranges from extremely small neurons to extremely large neurons.	How closely located neurons to one another. Overall density of entire layer.	Homogenous or heterogenous packing density within subfield. Clustering may occur at superficially, inferiorly, or in the middle within the pyramidal layer.	The neurons alignment or orientation to one another. Strong collinearity means neurons aligned in same direction.
Boundaries that this trait is useful in distinguishing:	CA1|CA2 ProS|CA1 ProS|Sub	CA1|CA2 ProS|CA1 ProS|Sub Sub|PreS PreS|ParaS	CA1|CA2 ProS|CA1 ProS|Sub Sub|PreS PreS|ParaS CA3|CA4	CA2|CA3 ProS|CA1 ProS|Sub PreS|ParaS	CA2|CA3 ProS|CA1 ProS|Sub PreS|ParaS
Boundaries that this trait is NOT useful in distinguishing:	CA2|CA3 CA3|CA4	CA2|CA3 CA3|CA4	CA2|CA3	Sub|PreS PreS|ParaS CA3|CA4 CA1|CA2 ProS|CA1	Sub|PreS CA3|CA4 CA1|CA2 ProS|CA1

**FIGURE 2:**
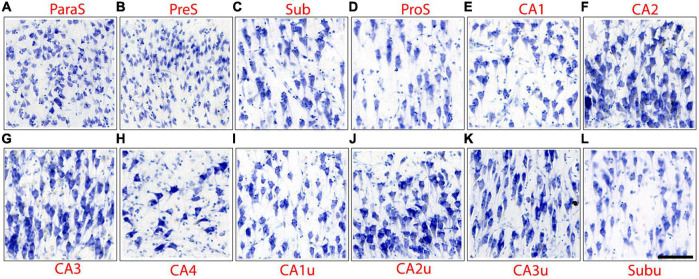
Microscopic images depict the neuronal characteristics, as outlined in the pentad protocol, of the hippocampal subfields. Images have been positioned so that the superior portion of the pyramidal layer is on the top of the image. Note that CA2 **(F)**, CA3 **(G)**, and CA4 **(H)** display intense chromophilia in their pyramidal neurons as well as medial counterparts CA2u **(J)** and CA3u **(K)**. Subfields CA1 **(E)**, Sub **(C)**, and ProS **(D)** show a moderate amount of chromophilia, as do their medial counter parts CA1u **(I)** and Subu **(L)**. ParaS **(A)**, PreS **(B)**, CA2, and CA3 present a dense packing density, while CA1, Sub, and CA4 display sparser neuronal populations. ParaS and PreS contain extremely small neurons in the superficial layer (layer II) while CA3 and CA4 in pyramidal layer exhibit the largest neurons. The CA2 and CA3 pyramidal neurons reveal the highest degree of collinearity.

**FIGURE 3:**
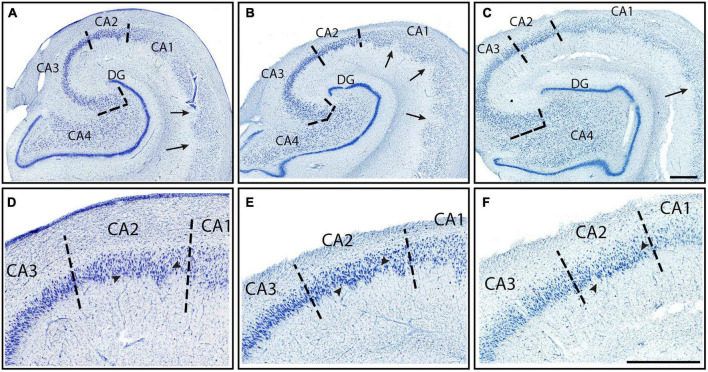
CA fields (CA1–CA3) from three different cases (Cases 12, 21, and 13, respectively) stained for Nissl substance. All slides at the level of the hippocampal body. **(A–C)** Macro scale photographs of three cases, and a zoomed in mesoscale image **(D–F)** showing CA1, CA2, and CA3 from the same Nissl stained section. Arrows point to the characteristic jaggedness found in CA1’s pyramidal layer. Note the change in staining intensity from CA1 and CA2, with CA1 showing lighter staining. CA1 contains smaller neurons than both CA3 and CA2, but CA2 and CA3 have similar neuron sizes and staining intensity. CA3’s neurons exhibit more collinearity than CA2. Arrowheads in panels **(D–F)** denote the clustering of neurons at the inferior edge of the pyramidal layer. Magnification bars, 1 mm.

**FIGURE 4:**
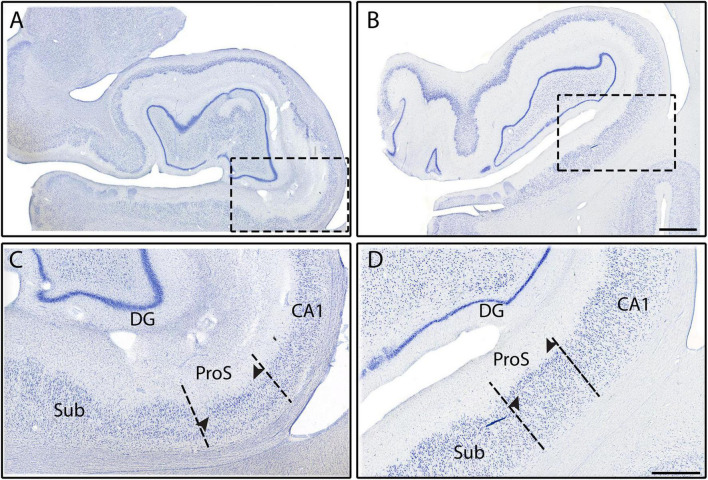
Subfields CA1, ProS, and Sub from two different cases (Cases 18 and 6, respectively). Histologic slides illustrated at the full DG level with photomicrographs of two respective Nissl stained sections **(A,B)**, and zoomed mesoscale images **(C,D)**. Black dashed rectangles correspond to the close-up view of panels **(C,D)**, showing Sub, ProS, and CA1. Arrowheads point to the superficial neuronal clustering in the ProS pyramidal layer. Note the smaller and more densely packed neurons in ProS. Magnification bar for panels **(A,B)**: 2 mm, for panels **(C,D)**: 1 mm.

**FIGURE 5:**
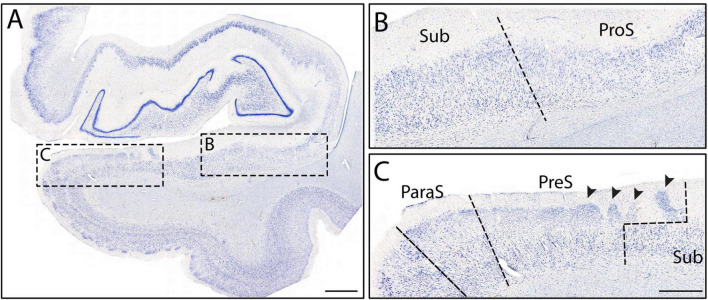
Macro **(A)** and mesoscopic views **(B,C)** of the subicular cortices from Case 3. Panel **(B)** shows ProS and Sub, while panel **(C)** shows ParaS, PreS, and Sub. Note the presubicular clouds overlay the distal part of Sub **(C)**, resulting in the staircase, or oblique boundary. Arrowheads point to the presubicular clouds. Medial to ParaS is the entorhinal cortex. Magnification bar for panel **(A)**: 2 mm, for panels **(B,C)**: 1 mm.

**FIGURE 6:**
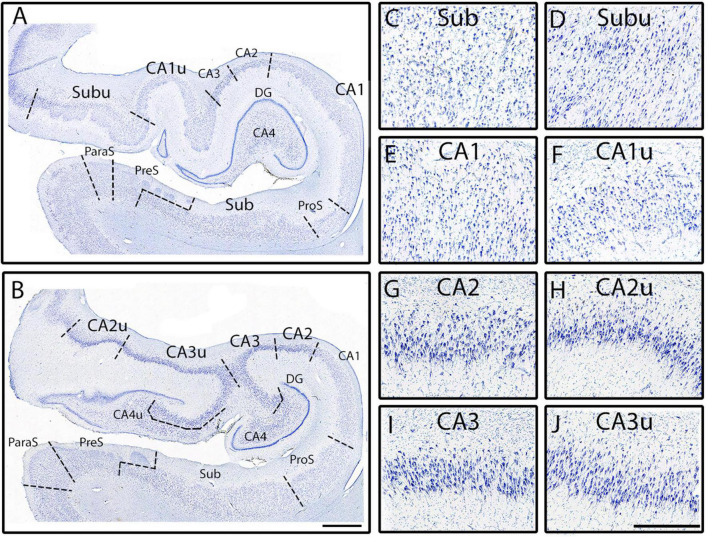
The medial CA subfields versus the lateral CA subfields. Macro view of fully parcellated Nissl stained section at the full DG **(A)** and x-region **(B)** levels from Case 12. Panels **(A,B)** show the macro views of the meso views of Sub **(C)**, Subu **(D)**, CA1 **(E)**, CA1u **(F)**, CA2 **(G)**, CA2u **(H)**, CA3 **(I)**, and CA3u **(J)**. Note the clustering of neurons in the middle of the pyramidal layer in Subu, and the more homogenously distributed pyramidal layer of Sub. CA1 and CA1u contain smaller and more lightly stained neurons, as well as less densely packed, than those in CA2, CA2u, CA3, and CA3u. Both CA2 and CA2u contain neurons that exhibit less collinearity than the neurons observed in CA3 and in CA3u. Finally, note how all medial subfields (denoted with a “u” at the end of the subfield name; far right panels) have smaller neurons than their lateral counterpart. Magnification bar for panels **(A,B)**: 2 mm, for panels **(C–J)**: 500 μm.

**FIGURE 7:**
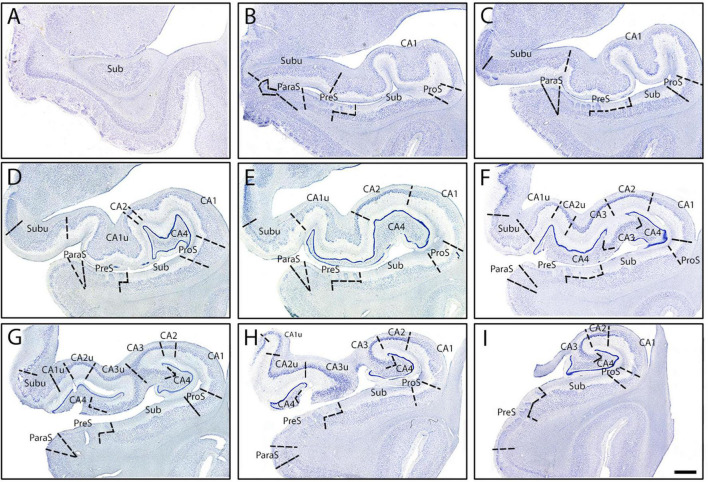
Parcellated human hippocampus from rostral to caudal levels from one case (Case 17). Panels **(A–I)** shows Nissl stained sections from each of the nine established levels (see Figure 1): genu **(A)**, genu-pes **(B)**, pes **(C)**, pes-DG **(D)**, full DG **(E)**, separated DG **(F)**, x-region **(G)**, uncus-body **(H)**, and body **(I)**, respectively. **(A)** The start of the hippocampus, typically only Sub, CA1, and rarely PreS is present. **(B)** ProS, ParaS emerge by this rostrocaudal point, as well as Subu. **(C)** As pes show a definite shape, similar subfield parcellation to panel **(B)**. **(D)** CA2, CA4, and CA1u appear, as well as the DG. **(E)** CA2 and CA4 both become larger, while Subu reduces in territory. **(F)** CA3 present, as well as CA2u. **(G)** Uncinate gyrus (and uncus) separates from the hippocampal body, and Subu and CA1u continue to get smaller. **(H)** ParaS and PreS shift more medially, Subu has disappeared. **(I)** ParaS and all medial subfields have ended. Note how subfields fluctuate in size (i.e., medial-lateral width) as hippocampus moves anterior to posterior. Magnification bar, 2 mm.

Note that certain borders have many traits that are listed as useful (e.g., CA1|CA2 border) – when identifying borders, multiple traits should be used collectively for peak accuracy. For borders where a trait is listed as not useful, other traits should be used when identifying that border. See Figures 2–7 for relevant Nissl stained sections and illustrations.

### 2.7. Application of the pentad protocol

This pentad protocol was applied to 13 unique hippocampal subfields (CA1, CA2, CA3, CA4, Sub, ProS, PreS, ParaS, CA1u, CA2u, CA3u, CA4u, and Subu) on all Nissl stained sections from each case (approximately 40 slides per case, depending on hippocampal size, and 22 cases). JA and EW performed the subfield parcellations. Subfields were compared to the other subfields on the same histologic slide for consistency. Figure 2 shows microscopic images for each 13 hippocampal subfields, and this figure especially highlights chromophilia, neuronal size, and collinearity. Packing density and cell clustering could also be inferred from this figure, but this should be done with caution since these two traits can change depending on the location within the pyramidal layer. Additional figures illustrate packing density (Figures 3A–C, 4C, D, 5B, C, 6A, B) and clustering (Figures 3D–F, 4C, D, 6A, B) best because these traits require a macroscopic view.

## 3. Results

Table 4 classifies the general observations and findings from the pentad protocol (chromophilia, neuron size, packing density, clustering, and collinearity) for all hippocampal subfields in our sample set. Detailed results of the pentad protocol will be described in the following sections. Each paragraph below compares a respective subfield to its immediate neighbors.

**TABLE 4:** Our hippocampal pentad protocol findings summarized for each subfield based on Nissl staining.

Pentad protocol	ParaS	PreS	Sub	ProS	CA1	CA2	CA3	CA4
Chromophilia	Light	Light	Moderate	Light	Light	Dark with gradient	Dark	Dark
Neuron size	Extremely small	Extremely small	Medium	Small	Medium	Large	Large	Extremely large
Packing density	Moderate	Dense	Sparse	Dense	Extremely sparse	Extremely dense	Extremely dense	Extremely sparse
Clustering	None	Clustered into clouds, and superior clustering within LPE	None	Often clustered superiorly	Occasional	Clustered inferiorly	None	None
Collinearity	None	Semi-collinear	None	Collinear	None	Semi-collinear	Collinear	Extremely noncollinear

A description of “None” means that that particular subfield did not have any observations of the given trait being described. See Figures 2–7 for relevant Nissl macro-, meso-, and microphotographs. LPE, lamina principalis externa.

### 3.1. Pentad findings of the CA fields

The first CA subfield that emerged anteriorly was CA1, which started slightly posterior to the subiculum. CA1 revealed the smallest and most lightly stained neurons among the CA fields (Figures 2E–G, 3). CA1 routinely showed a jagged or irregular appearance of its inner edge of the pyramidal layer (Figures 3A–C, arrows). Occasionally, CA1 had a clustering of neurons in the inferior portion of the pyramidal layer (Figures 3A–C, 7E–I). The neurons in CA1 were disorganized, showing a low degree of collinearity (Figure 2E). CA1 shared its borders with ProS and CA2. CA1 neurons were more sparsely packed than those in both ProS (Figure 4D) and CA2 (Figures 3D–F) and CA1 showed larger and more darkly stained neurons compared to ProS (Figures 2D, E, 4C, D). However, relative to CA2, CA1 had smaller and more lightly stained neurons (Figures 2E, F, 3D–F).

CA2 emerged at the level of the pes-DG (Figure 7D), slightly posterior to the beginning of the DG. As noted above, CA2 had larger, more darkly stained, and more densely packed neurons than CA1 (Figures 2E, F, 3D–F). CA2 also displayed clustering of neurons in its inferior portion of the pyramidal layer, a quintessential trait in CA2 (Figures 3D–F, arrowheads). The clustering in CA2 was more consistent and prevalent among different samples than in CA1; CA2’s clustering was present in nearly every histologic slide, whereas CA1’s clustering was present less than half of the time. Lastly, CA2 neurons were mostly disorganized (Figure 2F), though appeared to be slightly more collinear than CA1 neurons (Figure 2E). Collinearity was not as useful as the other traits in identifying the CA1/CA2 border since they were similar. Rather, the most distinguishing traits between CA1 and CA2 were chromophilia, clustering, and packing density on a mesoscale photomicrograph (Figures 3D–F) while microscopic images highlighted neuron size best (Figures 2E, F). Conversely, we identified the CA2/CA3 border with collinearity and clustering since CA2 and CA3 had similarly darkly stained, large, and densely packed neurons (Figures 2F, G). CA2 showed an inferior clustering, while CA3 does not (Figures 3D–F, arrowheads). Further, neurons in CA2 were much less collinear than CA3 neurons (Figures 2F, G).

CA3 contained large, highly chromophilic neurons, that were extremely densely packed (Figures 3D–F) and displayed a high degree of collinearity (Figure 2G). CA3 emerged posterior to CA2, around the separated-DG level (Figure 7F). There was no clustering of neurons in any portion of the pyramidal layer in CA3. Compared to CA4, CA3 neurons were slightly smaller (Figures 2G, H) but more densely packed (Figures 3A–C). Additionally, neurons in CA3 were extremely collinear, more collinear than any other subfield (Figure 2G). Respective neuron size, packing density, and collinearity were the most weighted traits between CA3 and CA4, since the subfields had similar staining intensities, and neither showed any neuron clustering (Figures 2G, H, 3A–C).

CA4 appeared slightly posterior to when the DG emerged and anterior to CA2, around the level of pes-DG. CA4 is located inside the hilus region and thus nestled within the DG. This subfield had the largest and least densely packed neurons of the CA fields but had similar staining intensities to CA2 and CA3 (Figures 2F–H, 3A–C). CA4 showed no clustering among its neurons (Figures 3A–C) and the neurons in CA4 exhibited the lowest collinearity of the CA subfields (Figure 2H).

### 3.2. Pentad findings of the subicular cortices

CA1 shares its other border with ProS, which first appeared between the genu and genu-pes levels. ProS featured lightly stained, small (Figure 2D), and densely packed neurons (Figures 4C, D, 5B). There was a clustering of neurons in the superior portion of the pyramidal layer within Prosubiculum (Figures 4C, D, arrowheads). This clustering in the superior region was not present in CA1, but it did occur, albeit rarely, in Sub. ProS also had smaller neurons (Figures 2C–E), greater packing density (Figures 4C, D, 5B), and higher collinearity than both CA1 and Sub (Figures 2C–E). Additionally, ProS was more lightly stained than CA1 and Sub (Figures 2C–E, 4C, D, 5B). Among hippocampal subfields, ProS exhibited the most pentad trait variability, ranging from very lightly stained and little clustering (Figure 5B) to darkly stained but prominent clustering (Figures 4C, D).

Subiculum, the first subfield to appear anteriorly in the hippocampus, was on the medial boundary of ProS. Sub occupied the most anterior portion of the hippocampus, approximately 250 μm coronally (Figure 7A) until CA1 emerged. Thus, anteriorly and prior to the arrival of ProS, CA1 and Sub shared a border and can be differentiated by their differences in chromophilia, packing density, and neuronal sizes. More specifically, Sub had darker stained, slightly more densely packed, and slightly smaller neurons than CA1. Of the subicular cortices, Sub showed the largest, most darkly stained (Figure 2C), and least densely packed neurons (Figures 4C, D, 5A–C). Neurons in Sub were not collinear relative to each other (Figure 2C). Sub very rarely exhibited neuronal clustering at its superior edge of the pyramidal layer.

Subiculum shares its medial border with PreS, which displayed lightly stained and substantially smaller neurons than Sub (Figures 2B, 5C). PreS emerged just before genu-pes level and contained both the presubicular clouds (Figure 5C, arrowheads) and the lamina principalis externa (LPE). The extremely small neurons in PreS were tightly packed (Figure 5C) and lightly stained (Figures 2B, 5C). PreS also displayed a moderate degree of collinearity (Figure 2B). The boundary between PreS and Sub was consistently oblique, often appeared as a “staircase boundary,” since superficially the presubicular clouds overlayed the distal portion of Sub (Figure 5C, arrowheads, Figures 7B–I). We observed that the presubicular clouds were present at all levels that the PreS was present, and typically contained two to four clouds per coronal section (Figures 7B–I). The shape of the individual presubicular clouds differed depending on the rostrocaudal level, more circular at anterior levels and rectangular at more posterior levels (Figures 7B–I).

Finally, the last and most medial subfield of the subicular cortices was ParaS. ParaS emerged slightly more posteriorly than PreS did, typically once the hippocampal fissure opened (roughly around genu-pes or pes region). The caudal end of ParaS was slightly posterior to the uncus-body level, approximately around the same location that the entorhinal cortex ended. ParaS revealed extremely small neurons that were densely packed (Figure 2A), although they were larger and more sparsely packed than those in PreS (Figure 5C). ParaS had a similar staining intensity to PreS. Finally, ParaS neurons were less collinear than PreS neurons (Figures 2A, B) but the collinearity trait was not weighted heavily or instrumental in parcellation for PreS and ParaS. We observed that ParaS layer II was not detached from the LPE, but rather, was often continuous with the LPE (Figures 5C, 7C–I). The most medial edge of ParaS bordered the entorhinal cortex, which had extremely large and darkly stained pyramidal and stellate neurons; a juxtaposition of the small neurons in ParaS and large neurons of entorhinal cortex (Figure 5C).

### 3.3. Pentad findings of the medial subfields

At the posterior hippocampus head levels (i.e., pes to x-region), certain subfields appeared twice on the same histologic slide. For instance, CA1 appeared not only in its dorsolateral location, but also on the medial (or uncal) portion of the hippocampal head near the uncus (Figures 6A, B). These medial subfields were parcellated and labeled as CA1u, CA2u, CA3u, CA4u, and Subu. We applied the pentad protocol to differentiate among the medial subfields and the protocol remained effective medially too. The differences in neuronal sizes within the medial subfields followed the same pattern observed in the lateral subfields. For instance, CA1u showed smaller neurons than CA2u and CA3u (Figures 2I–K, 6F, H, J), and CA2u and CA3u exhibited similar neuron sizes (Figures 6H, J). The medial subfields shared the same characteristics as their lateral counterparts, with two exceptions. First, each medial subfield displayed smaller neurons than its lateral counterpart (Figures 2C–L, 6C–J). For example, CA1u neurons were smaller than CA1 (Figures 6E, F) CA2u smaller than CA2, and so on. The second difference was between Sub and Subu. In approximately half of our cases, Subu showed neuronal clustering in either the middle or the dorsal portion of its pyramidal layer (Figures 6A, D). This clustering resembled that observed in inferior CA2 (Figures 3D–F) and superior ProS (Figures 4C, D). Aside from these two exceptions, the pentad findings were the same when comparing lateral and medial subfields.

## 4. Discussion

In this study, we established the first regimented and comprehensive Nissl based parcellation protocol for the adult human hippocampus. Our pentad protocol collates five cytoarchitectural and cellular attributes to differentiate the hippocampal subfields: chromophilia, neuron size, packing density, clustering, and collinearity. The traits in this protocol are flexible in their adaptation to individual variability, as individual variability is inevitable when parcellating human cortical tissue. We established nine distinct anterior-posterior levels, which provide anatomical orientation on the rostrocaudal axis. Further, our protocol allows for parcellation of the hippocampal head and medial subfields, which has been notoriously difficult. The establishment of this protocol will aid histologic parcellation of the human hippocampus and by reducing disagreements and resulting variability among different groups.

The macro-, meso-, and microscopic scales of the histologic sections deserve special discussion for the parcellation protocol. It is important to note that while Figure 2 highlights the differences in chromophilia, neuronal size, and collinearity among the subfields, not all pentad traits were optimally observed at the microscopic level. Rather, the use of multiple magnification levels (i.e., micro-, meso-, and macroscopic views) ensure each trait is properly identified. For instance, the traits chromophilia, neuronal size, and collinearity were observed best at the microscopic level, whereas the traits packing density and neuronal clustering were optimally visualized at the macro- or meso-views (Figures 3A–F, 4A–D, 5A–C). As such, evaluating tissue at multiple scales is imperative for accurate parcellation. In sum, the quintessential point is that the pentad protocol draws on all the above magnifications, macro-, meso-, and micro- to optimally identify the subfield boundaries.

The pentad traits and their assigned weightiness, or usefulness, depend on the subfields being evaluated. For example, the subfields CA2 and CA3 have similar packing density, chromophilia, and neuron size. Thus, the other two traits, collinearity, and clustering, must be used to distinguish subfields CA2 and CA3 (Figures 3D–F). Conversely, CA1 and CA2 can be differentiated by packing density, chromophilia, and neuron size (Figures 3D–F), but not by collinearity or clustering. This alternating approach also allowed this protocol to adapt to individual differences in subfields. For instance, in some cases ProS was differentiated by its smaller and less darkly stained neurons compared to CA1 and Sub (Figure 5B). However, in other cases, ProS was identified by its superior clustering of neurons and greater packing density relative to CA1 and Sub (Figures 4C, D), as the smaller lightly stained neurons were less noticeable. This difference in chromophilia among subfields may suggest variability in staining quality; however, this issue was avoided by examining subfields within the same slide, ensuring that the differing staining intensities were not due to staining quality. In sum, the weight of each pentad trait not only depends on specific subfields characteristics, but also on how the individual subfield presents in a particular case.

While this is the first Nissl based protocol of this magnitude, it is not the first publication in parcellating the subfields. Multiple groups have assigned subfields to some degree in photographs and figures using histology (Lorente de Nó, 1934; Rosene and Van Hoesen, 1987; Insausti and Amaral, 2004; Ding, 2013; Adler et al., 2014, 2018; Ding and Van Hoesen, 2015; Zilles et al., 2015; Insausti et al., 2017; Steve et al., 2017; de Flores et al., 2020; Palomero-Gallagher et al., 2020). It is important to emphasize that the present protocol sought to build upon the existing literature by not only providing a novel marker (collinearity), but also by evaluating the previous findings. Of note, we found that CA1 has smaller and less densely packed neurons compared to CA2 and CA3 (Figures 2E–G, 3C–E), in agreement with other groups (Lorente de Nó, 1934; Rosene and Van Hoesen, 1987; Duvernoy, 2005; Ding and Van Hoesen, 2015; Palomero-Gallagher et al., 2020). We also agree with other studies that it was the first CA field to emerge anteriorly (Duvernoy, 2005; Ding and Van Hoesen, 2015; Palomero-Gallagher et al., 2020). Additionally, in agreement with previous reports (Lorente de Nó, 1934; Rosene and Van Hoesen, 1987; Duvernoy, 2005; Ding and Van Hoesen, 2015; Palomero-Gallagher et al., 2020), we found that CA2 had large, darkly stained, and densely packed neurons. We also confirmed the existence of ProS and that its neurons were noticeably smaller than both CA1 and Sub neurons (Figures 4C, D; Lorente de Nó, 1934; Rosene and Van Hoesen, 1987; Ding, 2013; Ding and Van Hoesen, 2015). Further, we found that ProS typically showed a clustering of neurons superiorly in its pyramidal layer (Figures 4C, D; Rosene and Van Hoesen, 1987; Ding and Van Hoesen, 2015; Palomero-Gallagher et al., 2020). Finally, we also observed and included the stair-case, or oblique, boundary between Sub and PreS (Figures 7B–I), which other studies also observed (Rosene and Van Hoesen, 1987; Insausti and Amaral, 2004; Ding and Van Hoesen, 2015), and that PreS contained neurons that were smaller than those in ParaS (Figure 5C), which agreed with other’s observations as well (Ding and Van Hoesen, 2015; Insausti et al., 2017; Adler et al., 2018; Palomero-Gallagher et al., 2020).

The pentad protocol was applied not only for the lateral HP subfields, but also the medial ones such as CA1u, CA2u, CA3u, CA4u, and Subu. Notedly, our findings show that the neurons in the medial subfields were smaller than the ones in their lateral subfield agree with Ding and Van Hoesen’s (2015) findings. Differing findings were reported for presence of ProS in the medial location, such that Ding and Van Hoesen (2015) found ProS medially and we did not. Based on the pentad traits presented here, we observed subiculum, but not prosubiculum, in the uncal region. It is not clear whether this discrepancy is due to case demographic differences or rather due to the different methods (we used histochemistry while Ding and Van Hoesen used immunohistochemistry). Furthermore, Ding and Van Hoesen (2015) utilized layer characteristics while our protocol focused on cellular based traits, which could also account for this difference in findings.

While many of our findings agree with previous works, the hippocampal parcellation field is plagued with disagreements on the existence of certain subfields. Specifically, Adler et al. (2018) and Insausti and Amaral (2004) do not include CA4 as an individual subfield, whereas Palomero-Gallagher et al. (2020), Ding and Van Hoesen (2015), Duvernoy (2005), Rosene and Van Hoesen (1987), and Lorente de Nó (1934) include CA4 (or equivalent, CA3h). Adler et al. (2014) refers to CA4 as the “hilar region of DG.” However, Insausti and Amaral (2004) state unequivocally that CA4 should not be considered a separate subfield since it is not functionally or cytoarchitecturally different than its neighboring subfields and instead include it with CA3. The discrepancy leads to not only a semantic issue (i.e., different names but same structure), but also a lumping together of traits. Both scenarios create varying delineations (i.e., different definitions, and lumping CA3 and CA4 together) and confusion when parcellating the CA fields. Our data and other findings suggest that CA4 be labeled as its own subfield, separate from CA3, for two reasons. First, our data shows that CA4 is cytoarchitecturally different from its neighboring subfields, because it has larger and more sparsely packed, but less collinear, neurons than CA3 (Figures 2G, H). Second, Palomero-Gallagher et al. (2020) recently found that CA4 contains unique receptors compared to its neighboring subfields, suggesting functional differences. Another subfield that is often debated is ProS. Insausti and Amaral (2004) do not classify ProS as an individual subfield based on the same reasoning as CA4, a presumed lack of different functional and cytoarchitecture uniqueness when compared to its neighboring subfields. Palomero-Gallagher et al. (2020) found that ProS had different receptors than both CA1 and Sub, which suggests ProS differs in functionality from its neighbors. Additionally, a recent rodent study (Ding et al., 2020) found evidence of cell type differences between ProS and Sub, which further confirms the existence of ProS. Our data also highlights many cytoarchitectural differences between ProS and its immediate neighbors CA1 and Sub (Figures 2C–E, 4C, D, 5B), showing uniqueness and not merely a CA1 and Sub hybrid. These data suggest that ProS is an independent subfield and not a CA1 and Sub hybrid, which agrees with Palomero-Gallagher et al. (2020), Ding and Van Hoesen (2015), Rosene and Van Hoesen (1987), and Lorente de Nó (1934).

These disagreements in parcellation or nomenclature may be due to the limitations that have influenced previous reports, which we sought to overcome. Previous histology studies on the human hippocampi often lack boundaries lines drawn in the figures, an issue that does not plague non-human primate literature (Wang and Barbas, 2018). In the human literature, arrows or arrowheads were at the inferior or superior part (i.e., not both) of the pyramidal layer to denote the boundary (Lorente de Nó, 1934; Rosene and Van Hoesen, 1987). Duvernoy (2005) did not indicate where the boundary resides in the histology figures (for examples, see Duvernoy, 2005 Figures 7B, 8, 9, 65, and 66), but only in schemata (see Duvernoy, 2005 Figures 7A, 63, and 64). More recent publications have included concrete boundary lines for subfield parcellations (Insausti and Amaral, 2004; Ding, 2013; Adler et al., 2014; Ding and Van Hoesen, 2015; Palomero-Gallagher et al., 2020). However, some of these studies focused on the subicular cortices, and their scope did not include the entire hippocampus (Ding, 2013; Insausti et al., 2017). One study (Adler et al., 2014) based parcellations on one sample and one level (hippocampal body), and thus, did not provide examples of how these subfields vary among individuals or along the rostrocaudal axis. Conversely, Ding and Van Hoesen (2015) made great strides and provided detailed parcellation examples for multiple anterior-posterior levels of the hippocampus and illustrated several cases. Further, Ding and Van Hoesen (2015) demonstrated variability in the number of hippocampal pes among individuals by showing cases with two, three, or four digitations in the pes region. Individual variability is inevitable when investigating the architecture of the human brain. With each study, the field fills in more detail, and we sought to do just that by illustrating many cases at multiple magnification scales to highlight how subfield cytoarchitecture differs among cases.

Alternative stains beyond Nissl have also been used for subfield parcellation, which have provided valuable information for the subfields. It has been documented that the boundary between CA2 and CA3 is marked by the presence of mossy fibers in CA3 but not in CA2 (Lorente de Nó, 1934; Rosene and Van Hoesen, 1987; Duvernoy, 2005; Ding and Van Hoesen, 2015). A similar pattern was described in Ding and Van Hoesen’s (2015) and Rosene and Van Hoesen’s (1987) for ProS, which demonstrated a strong affinity for acetylcholinesterase, setting it apart from CA1 and Sub. However, while silver or enzyme stains provide valuable information for the presence of mossy fibers and acetylcholinesterase, respectively, the approaches do not provide other cytoarchitectural boundaries and add another adjacent slice to the series, making these stains difficult to rely on for mass parcellations (i.e., many reliable sections). As such, we sought to expand upon the immunohistochemistry work by providing a histochemistry based protocol (Nissl thionin), which is accessible, easy to use, shows both layers and cells, and is optimal for cytoarchitecture parcellation.

The limitations of the study include a long PMI (48 h) for one case, which could have affected staining quality. In addition, subclinical pathology may affect parcellation, though tau pathology in the hippocampus was limited and very isolated. We used preclinical cases (controls, Braak and Braak stages I and II) only and because of this, tau accumulation was primarily limited to the perirhinal and entorhinal cortex (Braak and Braak, 1991). Neurofibrillary tangles were isolated in the CA fields and Sub and thus contained minimal, if any, neuropathologic changes in the hippocampus. An additional limitation was that neuronal size was not quantitatively measured, but rather was only qualitatively estimated. A future study will have to tackle this quantitative endeavor. This work also requires evaluation using microscopy (i.e., high-magnification images) and experienced raters to distinguish bordering subfields, but it is our aim that this protocol educates the non-expert to the cytoarchitectural characteristics and illustrates boundaries histologically that can be extrapolated to neuroimaging. Finally, a limitation of the human histological literature is how blocking is done compared to in rodent brains, which follow precise 3D stereotaxic coordinates (Khazipov et al., 2015). As is the convention in the human brain, the hippocampus is typically hand blocked without a stereotaxic coordinate system (Insausti et al., 1995; Augustinack et al., 2005; Adler et al., 2014; de Flores et al., 2015; Steve et al., 2017). Blocking procedures in the human brain trail behind the stereotaxic approaches in the rodent brain, with exception (Buren and Maccubbin, 1962; García-Cabezas et al., 2007), and future studies will have to delve into this to standardize for human samples.

This scientific report contains several strengths. First and foremost, our study includes 22 cases, which is more cases than in the previously published parcellation reports. Second, our analysis was comprehensive; each of the 22 samples were stained for the entirety of the human hippocampus with over 850 stained Nissl sections. Third, we established a regimented protocol with five traits and nine rostrocaudal levels. Altogether, the present study demonstrates a detailed subfield segmentation of the human hippocampus, as it included a large number of cases and established nine distinct rostrocaudal levels. This novel protocol provides the hippocampal subfield segmentation field a concise yet multifaceted protocol, which aims to help standardize the histologic parcellation of the adult human hippocampus.

With the extensive interest in individual hippocampal subfield function and vulnerability, we aimed to build a detailed, histology-based parcellation protocol. The protocol’s ability to withstand individual, subfield, and anterior-posterior variability shows that this parcellation method offers clarity and consistency for neurohistology of the human hippocampus. It may ultimately provide a positive impact on the neuroimaging community. We implemented this novel pentad protocol to help reduce the differences found in hippocampus subfield parcellation among research groups and ultimately build cohesion. This protocol does so by providing a weighted guide with well-defined criteria, for researchers who study the hippocampus and its subfields. These findings provide a parcellation guide for the hippocampal subfields using the gold standard histology approach.

## Data availability statement

The original contributions presented in this study are included in the article/supplementary material, further inquiries should be directed to the corresponding author upon reasonable request.

## Ethics statement

The studies involving human participants were reviewed and approved by the Internal Review Board at the Massachusetts General Hospital. The patients/participants provided their written informed consent to participate in this study.

## Author contributions

EW and JA designed the study and wrote the manuscript. All authors contributed to the acquiring the data, staining, post processing of data, and revision of manuscript.
